# Increased Expression of the Mitochondrial Glucocorticoid Receptor Enhances Tumor Aggressiveness in a Mouse Xenograft Model

**DOI:** 10.3390/ijms24043740

**Published:** 2023-02-13

**Authors:** Aikaterini G. Karra, Ioannis Tsialtas, Foteini D. Kalousi, Achilleas Georgantopoulos, Evangelia Sereti, Konstantinos Dimas, Anna-Maria G. Psarra

**Affiliations:** 1 Department of Biochemistry and Biotechnology, University of Thessaly, Biopolis, 41500 Larissa, Greece; 2 Department of Pharmacology, Faculty of Medicine, University of Thessaly, Biopolis, 41500 Larissa, Greece

**Keywords:** glucocorticoid receptor, mitochondria, tumor growth, metabolism, pyruvate dehydrogenase, cancer

## Abstract

Mitochondria are important organelles for cellular physiology as they generate most of the energy requirements of the cell and orchestrate many biological functions. Dysregulation of mitochondrial function is associated with many pathological conditions, including cancer development. Mitochondrial glucocorticoid receptor (mtGR) is proposed as a crucial regulator of mitochondrial functions via its direct involvement in the regulation of mitochondrial transcription, oxidative phosphorylation (OXPHOS), enzymes biosynthesis, energy production, mitochondrial-dependent apoptosis, and regulation of oxidative stress. Moreover, recent observations revealed the interaction of mtGR with the pyruvate dehydrogenase (PDH), a key player in the metabolic switch observed in cancer, indicating direct involvement of mtGR in cancer development. In this study, by using a xenograft mouse model of mtGR-overexpressing hepatocarcinoma cells, we showed increased mtGR-associated tumor growth, which is accompanied by reduced OXPHOS biosynthesis, reduction in PDH activity, and alterations in the Krebs cycle and glucose metabolism, metabolic alterations similar to those observed in the Warburg effect. Moreover, autophagy activation is observed in mtGR-associated tumors, which further support tumor progression via increased precursors availability. Thus, we propose that increased mitochondrial localization of mtGR is associated with tumor progression possible via mtGR/PDH interaction, which could lead to suppression of PDH activity and modulation of mtGR-induced mitochondrial transcription that ends up in reduced OXPHOS biosynthesis and reduced oxidative phosphorylation versus glycolytic pathway energy production, in favor of cancer cells.

## 1. Introduction

Glucocorticoids (GCs) are steroid hormones that regulate a plethora of biological functions such as immune responses, growth, metabolism, differentiation, and apoptosis [1]. Glucocorticoid actions are mediated by their cognate receptors, the glucocorticoid receptors (GR), which, in their inactive form, are localized mainly in the cytoplasm in a form of GR-Heat shock protein (HSP) complex. Upon hormone binding, GR conformational alterations are induced, which are followed by release of the GR from the GR/HSP complex, GR translocation to the nucleus, dimerization, and binding to specific Glucocorticoid Response Elements (GREs), to regulate positively or negatively the expression of GR target genes. In addition, in the nucleus, GR via interaction with nuclear transcription factors, such as AP1 and NF-κB, regulate positively or negatively the transcription of target genes of those transcription factors [2]. Non-genomic actions of GCs, via membranous glucocorticoid receptors, have also been documented [3]. Moreover, the mitochondrial localization of GR in many cell types and its interaction with GREs in the mitochondrial DNA, and other mitochondrial localized regulatory molecules, transcription factors, and metabolic enzymes, such as Bcl-2, thioredoxin-2 (Trx2), HSP-60, and pyruvate dehydrogenase (PDH), have been reported, indicating a crucial role of GR in the regulation of mitochondrial biogenesis, energy production, immune responses, reactive oxygen species production, and apoptosis [4,5,6,7].

Mitochondria are the powerhouses of the cell, generating more than the 90% of the energy requirements, by way of oxidative phosphorylation in the respiratory chain. Mitochondria are also involved in metabolic processes such as heme and steroid hormone biosynthesis, urea production, beta-oxidation of fatty acids, and the Krebs cycle. Mitochondria are also involved in the reactive oxygen species (ROS) production, ROS detoxification by mitochondrial antioxidant enzymes, immunomodulation, Ca^2+^ homeostasis, cell differentiation, and apoptosis [8]. Thus, maintenance of mitochondrial function is considered vital for the maintenance of cellular physiology. Dysregulation of mitochondrial function is associated with ageing and many pathological conditions such as degenerative diseases and cancer progression [9,10,11,12,13]. According to the Warburg effect, metabolic reprogramming is an important cancer hallmark characterized by the activation of aerobic glycolysis, increased glutaminolysis, reduced energy production by oxidative phosphorylation, and macromolecule biosynthesis [14,15].

The mitochondrial localization of the glucocorticoid receptor and its involvement in the regulation of mitochondrial energy production and apoptosis prompted us to evaluate its potential involvement in tumorigenesis. For that purpose, tumors were produced in a xenograft model, upon injection of NOD.Cg-Prkdcscid/J (NOD/SCID) and NOD.Cg-Prkdcscid Il2rgtm1Wjl/SzJ (NSG) mice either with HepG2 cells overexpressing a mitochondrial-targeted green fluorescence protein (GFP)-GR fused protein or with HepG2 cells overexpressing a mitochondrial-targeted GFP, previously produced in our laboratory [5]. Evaluation of tumor growth rate and assessment of the biochemical pathway affected, via Western blot analysis and/or enzymatic activities measurements of certain metabolic enzymes and regulatory molecules, in total and mitochondrial extracts from the produced tumors, were performed. Our results revealed that mtGR increased tumor aggresiveness, an effect that, based on our experimental data, could be associated with the mtGR involvement in the regulation of mitochondrial OXPHOS biosynthesis, activation of autophagy, and modulation of mitochondrial metabolic enzymes synthesis and/or activity.

## 2. Results

### 2.1. mtGR Enhances the In Vivo Aggressiveness of HepG2 Cancer Cells

**Figure 1:**
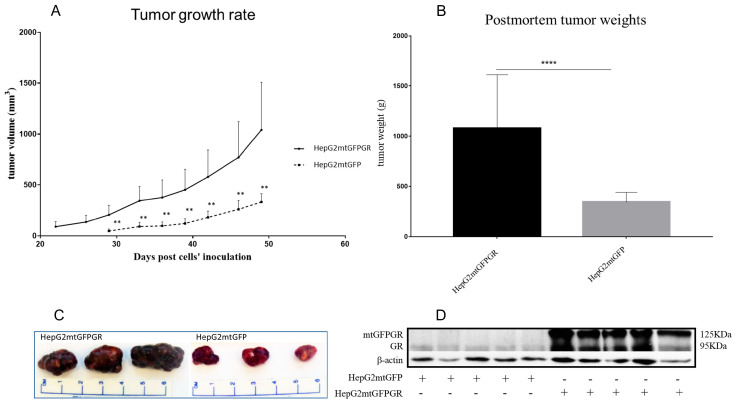
mtGR-induced increase in tumor progression in NOD/SCID mice inoculated with 1 × 10^6^ HepG2mtGFP or HepG2mtGFPGR cells. (**A**) The growth rates of the tumors in NOD/SCID mice and (**B**) postmortem weights of the tumors excised from the mice upon termination of the experiment. Each point shows the mean ± SD (*n* = 15–16, 8 mice per group), ** *p* < 0.01, **** *p* < 0.0001, compared to the HepG2mtGFP group. (**C**) Representative images of tumors from the two groups. (**D**) Western blot analysis of the expression of mtGR from five representative tumors from each group.

**Figure 2:**
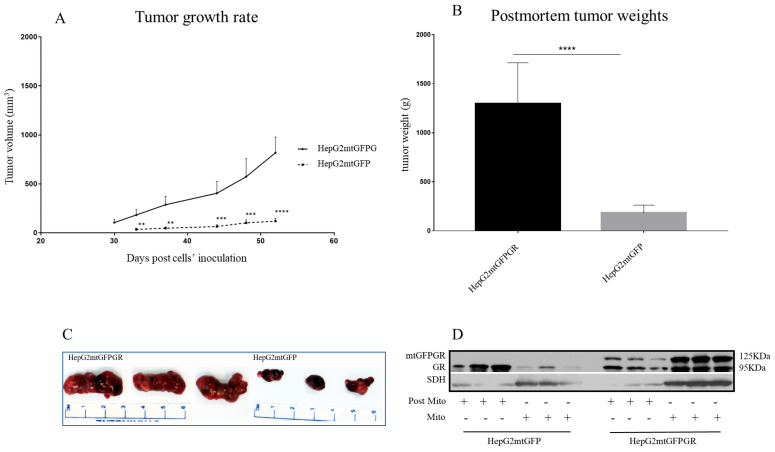
mtGR-induced increase in tumor progression in NSG mice inoculated with 1 × 10^5^ cells (HepG2mtGFP, HepG2mtGFPGR). (**A**) The growth rates of the tumors in NOD/SCID mice and (**B**) postmortem weights of the tumors excised from the mice upon termination of the experiment. Each point shows the mean ± SD (*n* = 10, 5 mice per group), ** *p* < 0.01, *** *p* < 0.001, **** *p* < 0.0001, compared to the HepG2mtGFP group. (**C**) Representative images of tumors from the two groups. (**D**) Western blot analysis of mtGR expression in Post-Mitochondrial (Post-Mito) and Mitochondrial (Mito) extracts from three representative tumors from each group.

**Figure 3:**
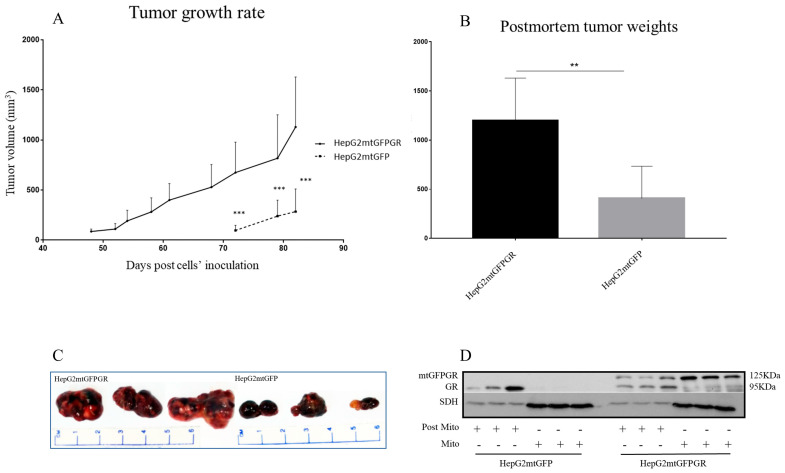
mtGR-induced increase in tumor progression in NSG mice inoculated with 1 × 10^4^ cells (HepG2mtGFP, HepG2mtGFPGR). (**A**) The growth rates of the tumors in NOD/SCID mice and (**B**) postmortem weights of the tumors excised from the mice upon termination of the experiment. Each point shows the mean ± SD (*n* = 8, 5 mice per group), ** *p* < 0.01, *** *p* < 0.001, compared to the HepG2mtGFP group. (**C**) Representative images of tumors from the two groups. (**D**) Western blot analysis of mtGR expression in Post-Mitochondrial (Post-Mito) and mitochondrial extract (Mito) from three representative tumors from each group.

As can be seen in Figure 1, Figure 2 and Figure 3 and Supplementary Figures S1 and S2, the overexpression of the GR in mitochondria (mtGR) turned HepG2 extremely aggressive. To study the role of mtGR in the aggressiveness of the HepG2 cells, we inoculated NSG and NOD/SCID mice with different densities of the HepG2mtGFPGR cells. As can be seen in Figure 1A, the control HepG2mtGFP cells (HepG2 cells stably transfected to express the mtGFP protein) resulted in palpable tumors in NOD/SCID mice at day 29 post-inoculation of the cells. On this day, 11 out of the 16 tumors inoculated (two bilateral tumors/mouse, eight mice pre group) were palpable in the mice that received the HepG2mtGFP cells as opposed to the 15 out of the 16 tumors for the HepG2mtGFPGR group. Moreover, the tumors expressing the mtGR showed much faster growth rates as compared to the controls (Figure 1A), which ultimately resulted in much larger tumors (over twofold larger), as can be seen by their corresponding postmortem weights and size (Figure 1B,C). To further confirm this difference and the effect of the mtGR on the tumorigenic capacity of the HepG2 cells, we injected with the same cells (HepG2mtGFP and HepG2 mtGFPGR) NSG mice that are more immunocompromised as they lack T, B, and NK-functional lymphocytes using two different cell inoculation densities, to study the potency of the tumorigenic features of both the control and the mtGR-expressing cells. As can be seen in Figure 2 and Figure 3 and Supplementary Figure S2, the lower inoculation density greatly affected the tumorigenic capacity of the HepG2mtGFP (control) cells in NSG mice. In mice injected with 10 × 10^5^ cells, tumors became palpable at day 33 post-inoculation of the cells and at day 72 for the mice inoculated with 10 × 10^4^ cells. Of interest in the latter case, only five out of the ten tumors were palpable on this day, while, at the termination of the experiment, there were still no signs of tumors in two sites of injection. On the contrary, the lower inoculation density resulted in less striking differences in the growth rate of the HepG2 cells expressing the mtGR. In the case of the mice that received the 10 × 10^5^ cells, all ten tumors became palpable on day 30, and for the mice that received the 10 × 10^4^ cells, all ten tumors became palpable on day 48 post-inoculation of the cells. In both cases, the postmortem examination of the tumors revealed that the mtGR-expressing tumors were 3 to 6-fold larger as compared to the non-expressing mtGR tumors (Figure 2B,C and Figure 3B,C). To confirm that the mtGR was overexpressed, representative tumors of all groups were processed after the end of the experiments and analyzed for the expression of the mtGR by Western blot (Figure 1D, Figure 2D, and Figure 3D). Quantitative analysis verified the mtGFPGR expression exclusively in HepG2mtGFPGR-associated tumors, as was expected, whereas no statistically significant differences in the endogenous GR protein levels, between the mtGFPGR- and mtGFP-expressing tumors, in both the total and the mitochondrial fractions, were detected (Supplementary Figure S3).

### 2.2. mtGR-Induced Tumorigenesis Is not Associated with mtGR-Associated Regulation of Apoptosis

**Figure 4:**
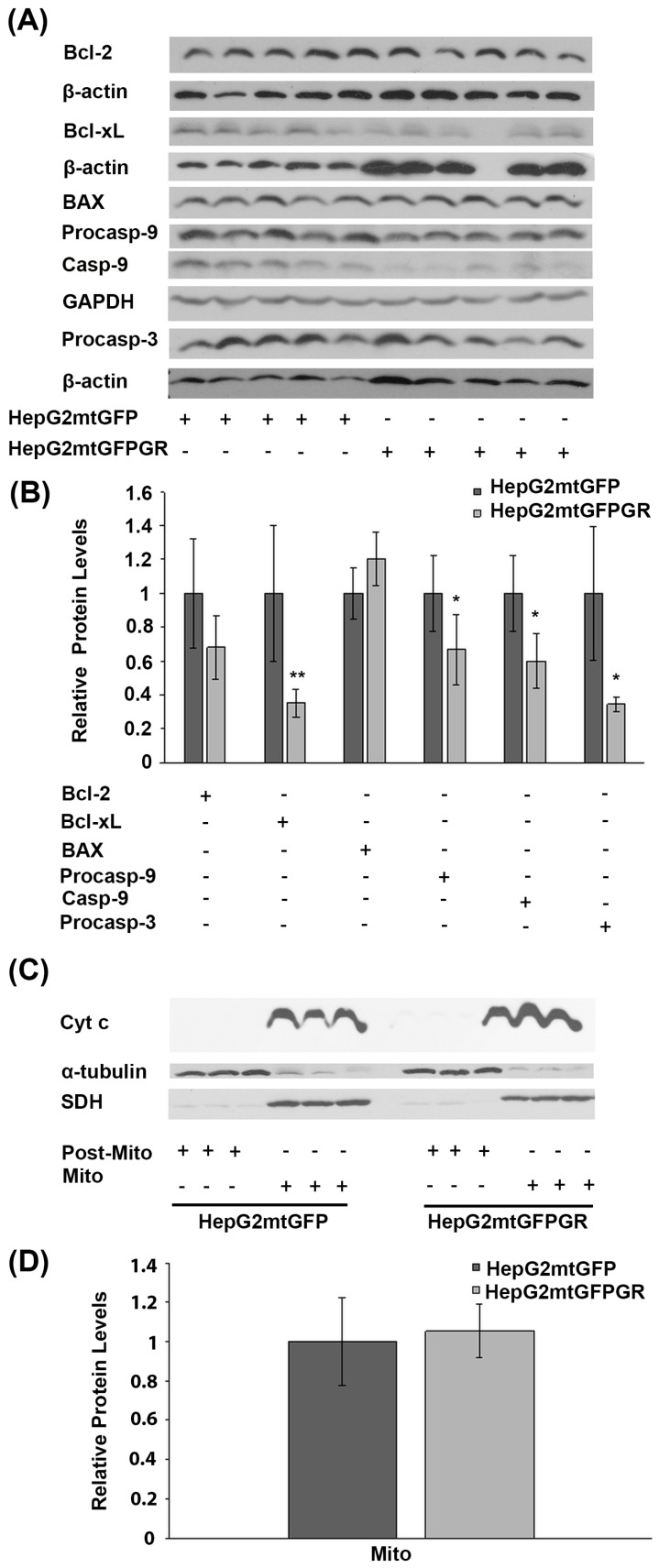
Assessment of the role of mtGR in the regulation of apoptosis, during tumor growth in human-to-mouse xenografts. (**A**) Western blot analysis of β-actin, glyceraldehyde 3-phosphate dehydrogenase (GAPDH), Bcl-2, Bcl-xL, BAX pre-cleaved caspase 9 (procasp-9), caspace-9, and pre-cleaved caspase 3 (procasp-3) in extracts from tumors produced in NOD-SCID mice, inoculated with HepG2mtGFPGR and HepG2mtGFP (control) cells. (**B**) Quantification of the results. Protein levels of apoptosis-associated molecules were normalized against β-actin or GAPDH protein levels. Protein levels in control tumors produced by HepG2mtGFP cells were set as 1. Relative protein levels are expressed as the mean ± S.D (*n* = 5), * *p* < 0.05, ** *p* < 0.01, compared to the protein levels in control tumors. (**C**) Western blot analysis of cytochrome c (Cyt c) in extracts from isolated mitochondria and post-mitochondrial fractions from tumors produced after injection with 1 × 10^4^ cells (HepG2mtGFP and HepG2mtGFPGR), in NSG mice. No Cyt c release in the post-mitochondrial fraction of both HepG2mtGFP- and HepG2mtGFPGR-associated tumors was detected. No differential expression of the mitochondrial Cyt c was observed. Mitochondrial SDH was used for the normalization of the mitochondrial protein levels, whereas β-tubulin was used for the normalization of the cytoplasmic protein levels. (**D**) Quantification of the results. Data are expressed as the mean ± S.D (*n* = 3), * *p* < 0.05, compared to control tumors produced by HepG2mtGFP cells.

Glucocorticoids are crucial regulators of mitochondrial-dependent apoptosis in a tissue-specific manner [16]. Specifically, mitochondrial GR is known to induce dose-dependent apoptosis in thymocytes and neuronal cells [17,18]. To elucidate the biochemical mechanism of mtGR involvement in tumor progression, the potential regulation of apoptosis by mtGR was assessed in the mtGFPGR-associated tumors compared to the control mtGFP ones, in NOD-SCID mice. To this purpose, protein levels of molecules associated with the mitochondrial-dependent apoptosis, such as procaspace-9, cleaved caspase-9, Bcl-2, Bcl-xL, BAX, and procaspace-3, were assessed in total extracts from tumors produced in NOD-SCID mice, applying Western blot analysis (Figure 4). As it is shown in Figure 4A,B, a statistically significant reduction in the anti-apoptotic Bcl-xL protein and the activated cleaved caspase-9 was observed in the mtGFPGR-associated tumors compared to the control ones. Yet, a reduction in cleaved caspase-9 protein levels is accompanied by a statistically significant reduction in non-activated procaspase-9 and -3 protein levels in the mtGFPGR-induced tumors compared to control ones, indicating no differential caspase 9 activation in the produced tumors of different origin. In line with this observation, no statistically significant differential expression of BAX and Bcl2 protein levels was observed in tumors generated by the two types of cell lines.

Differential activation of the mitochondrial-dependent apoptosis was also assessed in isolated post-mitochondrial and mitochondrial fractions from tumors produced in NSG mice, inoculated with 1 × 10^5^ HepG2mtGFP and HepG2mtGFPGR cells. As shown in Figure 3C,D, no release of the mitochondrial Cytochrome c (Cyt c) in the cytosolic fractions from tumor cells of both origins was observed. Moreover, no statistically significant differences in the protein levels of the mitochondrial Cyt c were observed, in tumors from both groups. Thus, we concluded that mtGR overexpression does not cause any differential effect on the induction of the mitochondrial-dependent apoptosis, upon tumor progression.

### 2.3. mtGR-Associated Increase in Tumor Aggressiveness Is Related to Autophagy Induction

**Figure 5:**
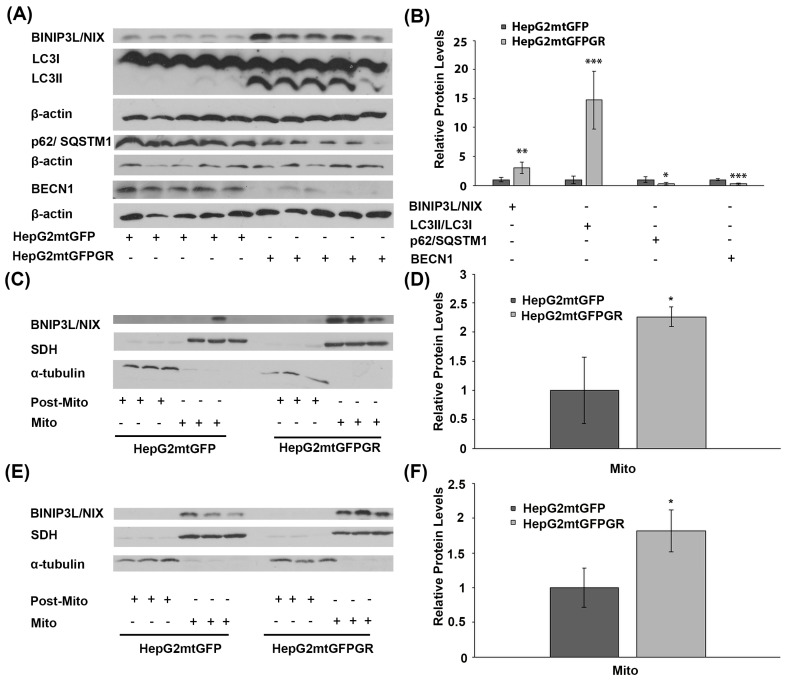
Involvement of mtGR in tumor aggressiveness via induction of autophagy. (**A**) Western blot analysis of β-actin, BINIP3L/NIX, LC3, p62, and BECN1 in extracts from tumors produced in NOD-SCID mice, inoculated with HepG2mtGFPGR or HepG2mtGFP (control) cells. (**B**) Quantification of the results. Relative protein levels were expressed as bands intensity normalized against the respective band’s intensity of β-actin. Relative protein levels in HepG2mtGFP tumors were set as 1. Proteins levels of LC3 are evaluated as the ratio LC3II/LC3I, normalized to β-actin protein levels. Data are expressed as mean ± S.D (*n* = 5), * *p* < 0.05, ** *p* < 0.01, *** *p* < 0.001, compared to relative controls. (**C**,**E**) Western blot analysis of BINIP3L/NIX protein levels in Post-Mitochondrial (Post-Mito) and Mitochondrial (Mito) extracts from tumors developed in NSG mice, upon inoculation with 1 × 10^4^ (**C**) or 1 × 10^5^ (**E**) HepG2mtGFP or HepG2mtGFPGR cells. (**D**,**F**) Relative BINIP3L/NIX protein levels were expressed as normalized against the respective succinate dehydrogenase (SDH) protein levels in mitochondrial extracts from tumors produced upon inoculation with 1 × 10^4^ (D) or 1 × 10^5^ (F) cells. Data are expressed as mean ± S.D (*n* = 3), * *p* < 0.05, compared to the respective controls. Protein levels of α-tubulin and SDH were assessed to verify mitochondrial purity and enrichment, respectively, and were used for the normalization of the results.

To explore the potential involvement of mtGR in tumor progression, via regulation of autophagy, comparative studies on the differential expression of autophagy-related molecules, such as BCL2/Adenovirus E1B 19 kDa protein-interacting protein 3-like (BNIP3L, BINIP3L/NIX), LC3II/LC3I (LC3B: microtubule-associated proteins 1A/1B light chain 3B), p62, and Beclin 1 (BECN1), were performed, applying Western blot analysis. Our results showed increased activation of autophagy in HepG2mtGFPGR tumors produced in NOD-SCID mice compared to that produced upon inoculation with HepG2mtGFP cells (Figure 5A,B). Specifically, protein levels of BINIP3L/NIX and LC3, the latter being evaluated as the ratio of conversion of LC3I (cytosolic form) to LC3II (LC3-phosphatidylethanolamine conjugate), were increased in the total extracts from mtGFPGR-associated tumors compared to the mtGFP-associated ones, indicating possible involvement of mtGR in the regulation of induction of autophagy [19]. Moreover, tumor suppressor proteins p62 and BECN1 [20,21] were reduced in HepG2mtGFPGR tumors, further supporting the hypothesis of induction of autophagy in the mtGR-associated tumors.

Induction of autophagy in tumors developed upon inoculation with mtGR-overexpressing HepG2 cells was also confirmed by Western blot analysis of BINIP3L/NIX in isolated post-mitochondrial and mitochondrial extracts from tumors produced in NSG mice, inoculated with 1 × 10^5^ (Figure 5C) and 1 × 10^4^ (Figure 5D) HepG2mtGFPGR cells. Protein levels of BINIP3L/NIX were approximately twofold higher in mitochondrial extracts from tumors grown in mice vaccinated either with 1 × 10^5^ or 1 × 10^4^ HepG2mtGFPGR cells, in comparison with the relative controls.

This observation is in agreement with results presented in Figure 5A,B. Moreover, as the increase in BINIP3L/NIX protein levels and decrease in p62 protein are associated with induction of mitophagy [22,23], these alterations could possibly indicate involvement of mtGR in induction of mitophagy, during tumor progression.

### 2.4. Regulation of OXPHOS Biosynthesis by mtGR upon Tumor Progression

**Figure 6:**
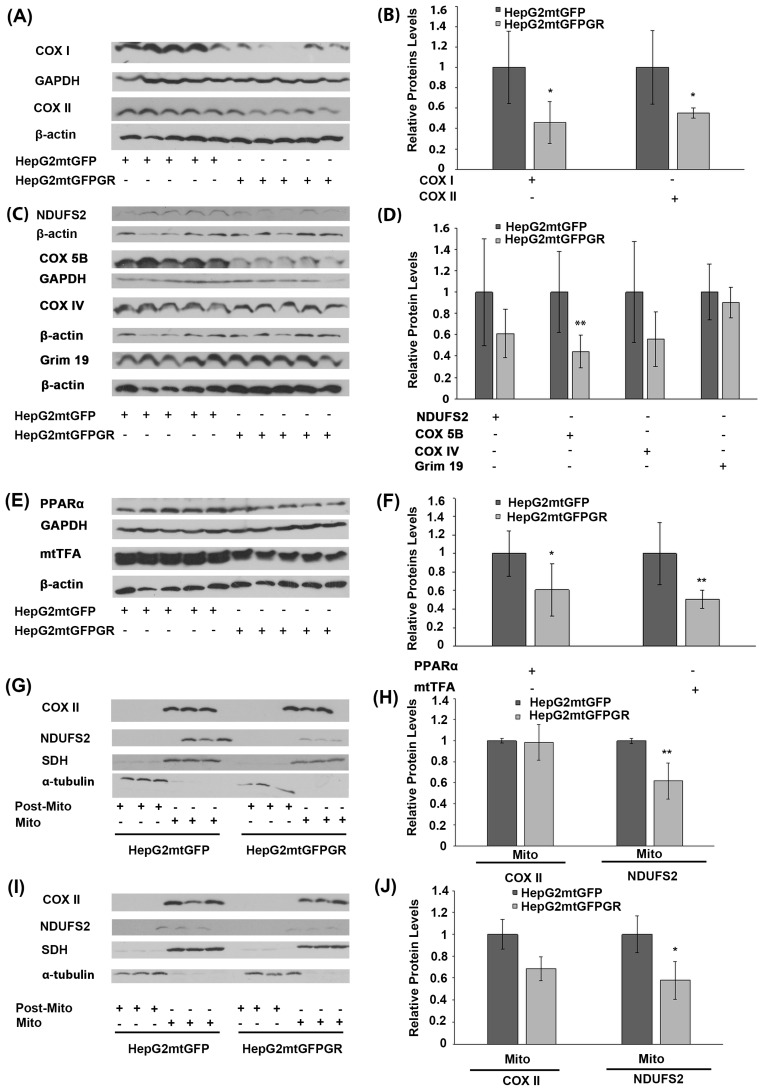
Regulation of OXPHOS biosynthesis by mtGR during tumor progression in human-to-mouse xenografts. Western blot analysis of (**A**) the mitochondrial-encoded OXPHOS subunits of COX I and COX II; (**C**) the nuclear-encoded OXPHOS subunits of NDUFS2, COX 5B, COX IV, and Grim 19; (**E**) the nuclear-encoded mitochondrial transcription-associated transcription factors, PPARα and mtTFA, in cell lysates of tumors produced in NOD-SCID mice, upon inoculation with 1 × 10^6^ HepG2mtGFPGR and HepG2mtGFP (control) cells. (**B**,**D**,**F**) Quantification of the results in (**A**,**C**,**E**), respectively. Protein levels were normalized against β-actin or GAPDH protein levels and data are expressed as mean ± S.D (*n* = 5), * *p* < 0.05, ** *p* < 0.01, compared to protein levels in control tumors, produced by HepG2mtGFP cells. (**G**,**I**) Western blot analysis of COX II and NDUFS2 protein levels in Post-Mitochondrial (Post-Mito) and Mitochondrial (Mito) extracts from tumors produced in NSG mice, inoculated either with 1 × 10^4^ (**G**,**H**) or 1 × 10^5^ (**I**,**J**) HepG2mtGFP and HepG2mtGFPGR cells. Protein levels of α-tubulin and SDH were assessed to verify mitochondrial purity and enrichment. Quantification of the results are presented in (**H**) and (**J**), respectively. Protein levels were normalized against the protein levels of the mitochondrial SDH. Data are expressed as mean ± S.D (*n* = 3), * *p* < 0.05, ** *p* < 0.01, compared to controls. Relative protein levels in mitochondria from tumors produced by HepG2mtGFP cells were set as 1.

Glucocorticoids via their cognate receptors, the glucocorticoid receptors, and particularly both via the nuclear and the mitochondrial localized ones, regulate mitochondrial transcription, and, thus, OXPHOS synthesis and energy production [5,24]. To assess whether mtGR’s effect on mitochondrial bioenergetics also has an impact on tumor progression, protein levels of the mitochondrial- (Figure 6A,B) and nuclear- (Figure 6C,D) encoded subunits of OXPHOS enzymes, as well as protein levels of nuclear-encoded transcription factors (Figure 6E), crucial for mitochondrial biogenesis, were evaluated in extracts from tumors produced in NOD-SCID mice, upon inoculation with 1 × 10^6^ HepG2mtGFPGR and HepG2mtGFP cells (Figure 6). As shown in Figure 6A,B, mitochondrial-encoded OXPHOS subunits of cytochrome c oxidase, subunit I (COX I), and II (COXII) were reduced in mtGFPGR-associated tumors compared to the mtGFP-associated ones. Similarly, a reduction in the nuclear-encoded OXPHOS subunits of complex I: NADH dehydrogenase [ubiquinone] iron-sulfur protein II (NDUFS2), NADH dehydrogenase [ubiquinone] 1 alpha subcomplex subunit 13 (GRIM 19 or NDUFA13), and Complex III: COX5B and COXIV (Figure 6C,D) was observed. Moreover, in line with OXPHOS reduction, a reduction in transcription factors involved in the regulation of the mitochondrial transcription and mitochondrial biogenesis, namely the peroxisome proliferator-activated receptor alpha (PPARα) and the mitochondrial transcription factor A (mtTFA), was observed in mtGFPGR-associated tumors compared to the control ones (Figure 6E,F).

In accordance with these observations, reduction in the mitochondrial-encoded COX II and the nuclear-encoded NDUFS2 protein levels was also confirmed in isolated mitochondria from HepG2mtGFPGR tumors produced in NSG mice, upon inoculation with 1 × 10^4^ (Figure 6G,H) and 1 × 10^5^ cells (Figure 6I,J), compared to controls. Interestingly, an increased number of cells (1 × 10^5^ versus 1 × 10^4^) used for tumor generation was followed by an increased reduction in the mitochondrial COX II level. Similarly, an increased reduction in the nuclear-encoded NDUFS2 protein levels was also observed, although to a lower extent.

### 2.5. Role of mtGR in the Regulation of Krebs Cycle during Tumor Progression

**Figure 7:**
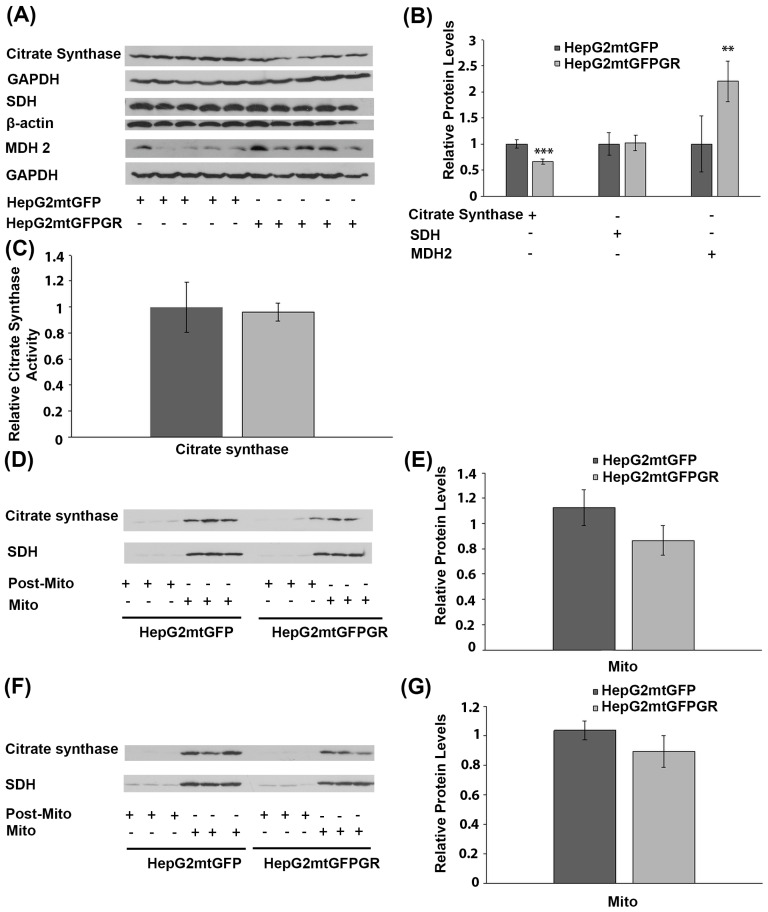
Effect of mtGR on Krebs cycle during in vivo tumor progression. (**A**) Western blot analysis of β-actin, GAPDH, citrate synthase, SDH, and MDH2 in extracts from tumors produced in NOD-SCID mice, inoculated with HepG2mtGFPGR and HepG2mtGFP (control) cells. (**B**) Quantification of the results. Protein levels of Krebs cycle enzymes were normalized against β-actin or GAPDH protein levels. Data are expressed as mean ± S.D (*n* = 5), ** *p* < 0.01, *** *p* < 0.001. (**C**) Relative citrate synthase activity in mtGFPGR-associated tumors was expressed in comparison to the citrate synthase activity in mtGFP-associated tumors. Data are expressed as mean ± S.D (*n* = 3). Citrate synthase activity in mtGFP-associated tumors was set as 1 (control). (**D**,**F**) Western blot analysis of citrate synthase protein levels in isolated mitochondrial (Mito) and post-mitochondrial (Post-Mito) extracts from tumors produced in NSG mice, inoculated with 1 × 10^4^ (**D**) or 1 × 10^5^ (**F**) HepG2mtGFP and HepG2mtGFPGR cells, respectively. (**E**,**G**) Quantification of the results in (**D**,**F**), respectively. SDH protein levels were used for the normalization of the results. Data are expressed as mean ± S.D (*n* = 5), ** *p* < 0.01, *** *p* < 0.01.

To investigate the possible effect of mtGR overexpression on Krebs cycle regulation, during tumor progression, protein levels of citrate synthase, succinate dehydrogenase (SDH), and malate dehydrogenase-2 (MDH 2), an isoform found to be increased in cancer cells [25,26], were analyzed in total extracts from tumors produced in NOD-SCID mice, upon inoculation with 1 × 10^6^ HepG2mtGFPGR or HepG2mtGFP cells (Figure 7A,B). No alteration in SDH protein levels was detected between the two types of tumors. Citrate synthase protein levels showed a moderate reduction, by approximately 20%, in tumors produced by HepG2mtGFPGR cells, compared to control tumors. On the contrary, protein levels of MDH 2 were increased by 2–3-fold in mtGFPGR-associated tumors, compared to controls. The possible effect on citrate synthase activity was also evaluated by applying a colorimetric enzymatic assay (see Materials and Methods section) in cell extracts from tumors produced in NOD-SCID mice upon inoculation with HepG2mtGFPGR or HepG2mtGFP cells. No statistically significant differences in citrate synthase activity **(**Figure 7C) were observed between the two group of tumors, in line with the results from Western blot analysis, showing slight alterations in citrate synthase protein levels. This result was also confirmed in isolated mitochondria from tumors produced in NSG mice, inoculated with 1 × 10^4^ (Figure 7D) or 1 × 10^5^ (Figure 7E) HepG2mtGFP or HepG2mtGFPGR cells.

### 2.6. Effect of mtGR on PDH Activity during Tumor Progression

**Figure 8:**
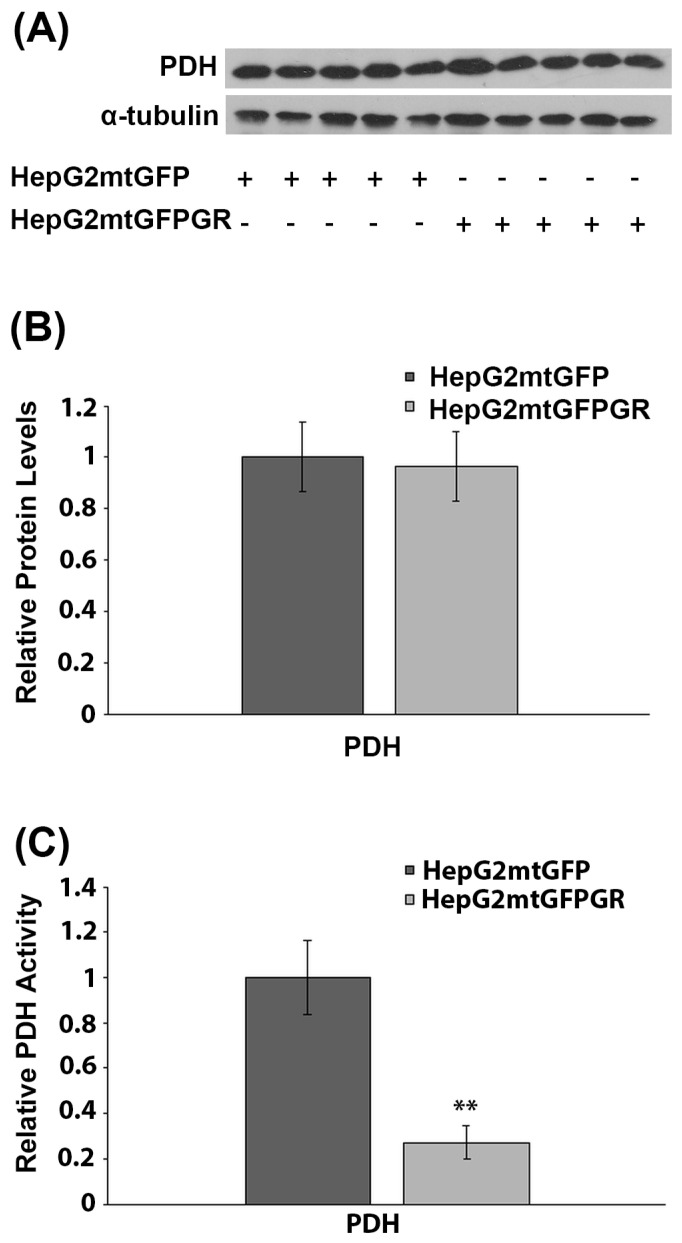
Mitochondrial GR modulates PDH activity during in vivo tumor progression. (**A**) Western blot analysis of PDH in extracts from tumors produced in NOD-SCID mice, upon inoculation with HepG2mtGFPGR or HepG2mtGFP (control) cell lines. (**B**) Quantification of the results. PDH protein levels were normalized against α-tubulin protein levels. Data are expressed as mean ± S.D (*n* = 5). (**C**) Relative enzymatic activity of PDH is expressed as enzymatic activity of PDH in HepG2mtGFPGR-associated tumors, compared to the PDH activity in HepG2mtGFP-associated ones. Enzymatic activity of PDH in HepG2mtGFP tumors was set at 1. Data are expressed as mean ± S.D (*n* = 3), ** *p* < 0.01, compared to controls.

PDH is a crucial regulator of mitochondrial energy metabolism. Inactivation of PDH is associated with the metabolic reprogramming observed during carcinogenesis [27]. More interestingly, PDH was recently found to interact with mtGR in the mitochondrial environment [6]. To investigate the possible involvement of mtGR in the regulation of PDH activity and, thus, of tumor progression, the protein levels and enzymatic activity of PDH were evaluated in total extracts from tumors produced in NOD-SCID mice, upon inoculation with 1 × 10^6^ HepG2mtGFPGR or HepG2mtGFP cell lines. Western blot analysis (Figure 8A,B) showed no effect on PDH protein levels, by mtGFPGR overexpression, in the developed tumors. On the contrary, evaluation of PDH activity, in total extracts from tumors generated upon injection with the HepG2mtGFPGR cells, showed an approximately fourfold reduction in PDH enzymatic activity compared to the control tumors, developed by the HepG2mtGFP cells (Figure 8C). This observation possibly indicates that the recently documented mtGR/PDH interaction [6] may affect PDH activity, in favor of tumor growth and progression, enhancing ultimately the aggressiveness of the tumor.

### 2.7. Effect of mtGR on Glucose Metabolism and Inflammation

**Figure 9:**
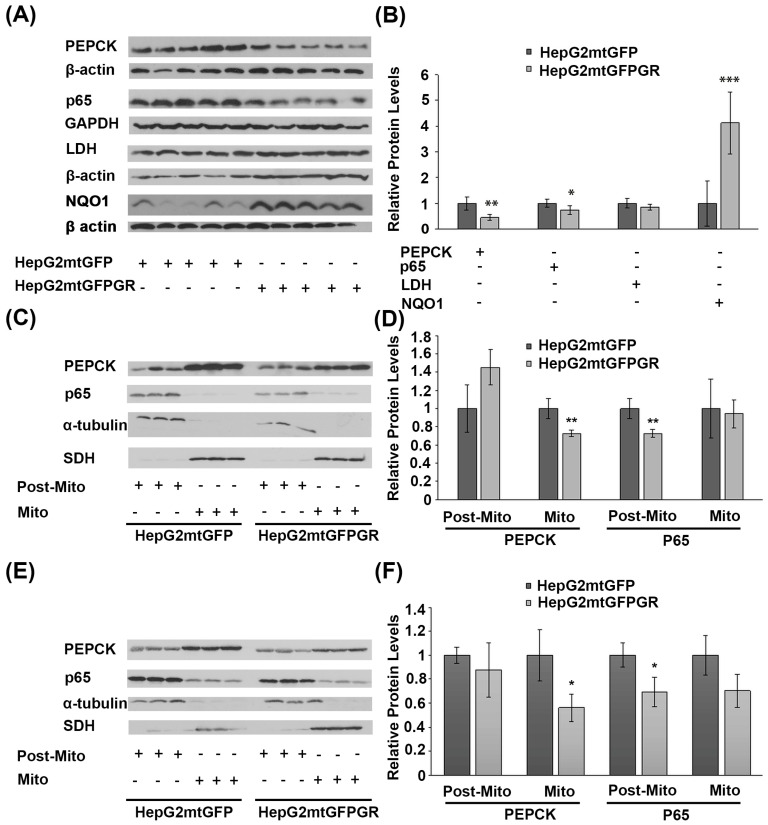
Increased mitochondrial localization of GR affects protein levels of glucose metabolism enzymes and inflammatory factors in cancer cells derived from xenografted tumors. (**A**) Western blot analysis of PEPCK, p65, LDH, and NQO1 in total extracts from tumors produced in NOD-SCID mice, inoculated with HepG2mtGFPGR or HepG2mtGFP (control) cells. (**B**) Quantification of the results. Protein levels of β-actin or GAPDH protein levels were used for the normalization of the results. Data are expressed as mean ± S.D (*n* = 5), * *p* < 0.05, ** *p* < 0.01, *** *p* < 0.001, compared to controls. (**C**,**F**) Western blot analysis of PEPCK and p65 in the post-mitochondrial (Post-Mito) and mitochondrial (Mito) extracts from tumors produced in NSG mice, inoculated with 1 × 10^4^ (**C**,**D**) or 1 × 10^5^ (**E**,**F**) HepG2mtGFP or HepG2mtGFPGR cells. SDH and α-tubulin were used for the normalization of protein levels in mitochondrial and post-mitochondrial extracts. Relative protein levels were expressed as mean ± S.D (*n* = 3), * *p* < 0.05, ** *p* < 0.01, compared to controls.

Glucose metabolism via aerobic glycolysis is a hallmark feature of cancer cells to meet their high energy demands and precursors availability for tumor growth and progression [28]. On the other hand, prevention of cancer development relies on a strong immune system and antioxidant defense mechanisms [29,30]. Considering the anti-inflammatory actions of glucocorticoids and their crucial role in the regulation of glucose metabolism, the possible outcome of mtGR overexpression in the regulation of the protein levels of the gluconeogenic enzyme phosphoenolpyruvate carboxykinase (PEPCK), the glycolytic lactate dehydrogenase (LDH), the p65 subunit of the inflammatory nuclear factor NF-kappa-B, and the NAD(P)H quinone oxidoreductase (NQO1) was assessed, in the developed tumors. As shown in Figure 9A,B, Western blot analysis showed a reduction in PEPCK and p65 protein levels in total extracts from tumors developed upon inoculation with 1 × 10^6^ HepG2mtGFPGR cells, in NOD/SCID mice, compared to the relative controls. No changes in the protein levels of LDH were observed, whereas an increase in the antioxidant enzyme NQO1 was detected. A reduction in the mitochondrial PEPCK and the cytosolic p65 subunit of NF-κB was also confirmed in isolated mitochondria and post-mitochondrial fractions, respectively, from the mtGR-associated tumors, developed in NSG mice, upon inoculation with 1 × 10^4^ (Figure 9C,D) or 1 × 10^5^ (Figure 9E,F) cells, compared to controls.

## 3. Discussion

Maintenance of mitochondrial energy production via oxidative phosphorylation and preservation of PDH activity are crucial for the prevention of tumor progression [31,32]. Thus, mitochondrial GR via its direct involvement in the regulation of OXPHOS biosynthesis and energy production [4,5] and via its potential involvement in the regulation of PDH activity, as a component of the PDH complex interacting proteins [6], could constitute a crucial regulatory factor in cancer development.

To validate this hypothesis, we studied the role of mtGR in tumorigenesis, using human-to-mouse xenografts that were developed in NOD-SCID and NSG mice. Toward this aim, we inoculated mice either with HepG2 cells stably transfected to overexpress a mitochondrial-targeted GFPGR or a mitochondrial-targeted GFP protein (served as control) [5]. Our results revealed increased tumorigenic potency in terms of take rate and tumor growth rate by mtGFPGR-overexpressing HepG2 cells compared to controls. Interestingly, the mtGFPGR-overexpressing HepG2 cells retained a very aggressive phenotype as they were able to develop tumors with a high growth and take rate even at low inoculation densities unlike the control cells (i.e., the mtGFP-expressing HepG2 cells). This observation substantiates a crucial role of the mtGR in tumor progression under the experimental conditions tested herein.

mtGR-induced tumorigenesis was accompanied by alterations in mitochondrial energy metabolism enzymes synthesis and activity. Thus, reduced expression of the mitochondrial-encoded COX I and COXII OXPHOS subunits in the mtGR-associated tumors was observed. This effect could possibly lead to a reduction in energy production via mitochondrial oxidative phosphorylation, in favor of cancer cells growth and survival, in line with the Warburg effect [14]. A reduction in mitochondrial-encoded OXPHOS subunits was also accompanied by a reduction in the expression of nuclear-encoded OXPHOS subunits (COX5B, COXIV, GRIM 19, NDUFS2) and in a reduction in the expression of nuclear-encoded transcription factors, which act as crucial regulators of mitochondrial transcription, such as PPARα and mtTFA. These observations validate the hypothesis of mitochondrial–nuclear communication in OXPHOS gene expression, a process orchestrated, among others, by steroid hormones, including glucocorticoids, via their cognate receptors [4]. The only exception was the COX15 gene expression, which was found to be increased by tumors formation. This observation is in line with previous studies, demonstrating a potential role of COX15 as a novel oncogene [33,34]. A reduction in OXPHOS enzyme biosynthesis in the mtGR-associated tumorigenesis is opposed to observations from ours and other laboratories, revealing an induction of mitochondrial transcription by mtGR in several type of cells, including neuronal and hepatocarcinoma cells, in culture [5,18,35,36,37]. Reversal of the mtGR effect on mitochondrial transcription, during tumorigenesis, is an interesting finding and could be attributed to alterations in mtGR protein–protein interactions, occurring during tumorigenesis. Changes in such mtGR interactions may cause alterations in the structural conformation of mtGR that subsequently could lead to modulation of its activity, ending up in inhibition of the mitochondrial transcription. These effects could take place in parallel to tumorigenesis-associated alterations in mRNA translational mechanisms [38,39] that could finally lead to suppression of OXPHOS biosynthesis.

In the same frame, a reduction in PDH activity, in mtGR-associated tumors, was observed, which was not associated with alterations in PDH protein levels. We have recently demonstrated an interaction of mtGR with PDH [6]. We hypothesize that this interaction could possibly lead to conformational changes in PDH that subsequently could modulate its accessibility to PDH kinase or PDH phosphatase, and thus, alter its enzymatic activity, by keeping it to its phosphorylated inactive state, in favor of tumor progression [31].

The metabolic pathway of the citric acid cycle was also found to be affected, in tumors overexpressing mtGR. Thus, a moderate reduction in citrate synthase expression was observed in mtGR-expressing tumors. Reduced citrate synthase is proposed to be linked to tumor malignancy via the Warburg effect [40]. A reduced participation of the Krebs cycle and oxidative phosphorylation in energy production favors tumor growth. In addition, an increase in isoform 2 of malate dehydrogenase was observed in mtGR-expressing tumors in line with previous observations, demonstrating increased malate dehydrogenase-2 in hepatocellular carcinoma and proposing MDH-2 as a new cancer biomarker [25,26].

To gain further insights into the metabolic alterations induced by mtGR-overexpression in cancer cells, protein levels of glycolytic enzymes such as lactate dehydrogenase and the gluconeogenic PEPCK enzyme were assessed. As was expected, the protein levels of the gluconeogenic mitochondrial PEPCK enzyme were reduced, whereas no alterations in LDH protein levels were observed. Thus, gluconeogenesis was suppressed against the glycolytic pathway activation, as indicated by the PEPCK reduction and the increase in NQO1 protein levels. NQO1 is also considered as a source of NAD regeneration to fuel the glycolytic pathway. Thus, an increase in NQO1 protein level could ensure NAD availability, as an alternative to the LDH source for NAD regeneration [41], to sustain ATP production via the glycolytic pathway, even in the presence of oxygen.

Glucocorticoids via their cognate receptors are known to induce apoptosis in a cell-type-specific manner. Nevertheless, as was expected, no activation of apoptosis was observed in tissue extracts originated from the two group of tumors. Interestingly, an alteration in autophagy markers such as a decrease in p62 and increase in LC3II/LC3I ratio and BINIP3L/NIX protein levels was observed, in accordance with previous observations showing autophagy activation during tumorigenesis [42], also indicating a Warburg-effect-associated induction of mitophagy, as suggested by the observed reduced p62 protein levels [43].

Because tumors are composed of different types of cells, the above-mentioned metabolic adaptations, triggered by mtGR overexpression, refers to resultant reactions of a heterogenous cancer and host cells populations varying at a cellular and metabolic level [14]. Thus, mtGR overexpression, under hypoxia conditions, triggers the aggressiveness of tumorigenesis not only via the enhancement of tumor proliferation rate but also via the possible establishment of different interactions of cancer cells with different stromal cells and/or cancer cells subpopulations, affecting metabolic symbiosis of glycolytic and oxidative cancer cells and/or activation of autophagy and, thus, metabolic parasitism. This is, indeed, an interesting hypothesis that needs to be validated by further studies. Under physiological conditions, mtGR could possibly promote different cellular interactions with neighboring cells, contributing to the maintenance of cellular and tissue physiology. In this context, glucocorticoids, via the coordination of the nuclear and the mitochondrial glucocorticoid receptor, may support non-cancerous cells survival, and possibly restrain the development of the Warburg effect, by the activation of the mitochondrial GR translocation, OXPHOS biosynthesis, oxidative energy production, and regulation of apoptosis, as has been previously described [4,18,35,36,37]. This hypothesis, if true, substantiates the fact that many cancer patients receive GR binding drugs to offset the side-effects of chemotherapy [44].

To conclude, mitochondrial glucocorticoid receptor overexpression contributes to the enhancement of HepG2 aggressiveness in the induction of tumor growth and progression, which is accompanied by reduced OXPHOS biosynthesis, reduction in PDH activity, and alterations in the Krebs cycle and glucose metabolism. Moreover, autophagy activation is observed in mtGR-associated tumors, which could further support tumor progression via increased precursors availability. These metabolic alterations resemble those observed in the Warburg effect. Considering previous observations substantiating the role of mtGR in the regulation of mitochondrial transcription and its interaction with PDH, we propose that during tumorigenesis, increased mitochondrial localization of mtGR caused the aggressiveness of tumor growth and progression possible via alterations in the mtGR/PDH protein complex, which could lead to suppression of PDH activity and modulation of mtGR-induced mitochondrial transcription that ends up in reduced OXPHOS biosynthesis and reduced oxidative phosphorylation versus glycolytic pathway energy production, in favor of tumor progression. Our results uncover the mitochondrial glucocorticoid receptor as a novel crucial regulatory factor in tumor progression, highlighting its importance as a potent therapeutic target for cancer treatment and prevention.

## 4. Material and Methods

### 4.1. Chemicals

Dulbecco’s modified Eagle medium (DMEM) and fetal bovine serum (FBS) were obtained from Thermo Fischer Scientific (GmbH, Frankfurt, Germany). Complete protease inhibitors cocktail was obtained from Roche (Mannheim, Germany). Acrylamide-bis acrylamide and Bradford reagent were purchased from Bio-Rad Laboratories (Athens, Greece). Molecular weight protein markers were purchased from Fermentas (Thermo Fischer Scientific, GmbH, Frankfurt, Germany). All other chemicals were purchased from Sigma-Aldrich (St. Louis, MO, USA). Details on the source of the chemicals used are presented in Supplementary Table S1.

### 4.2. Antibodies

GR-specific affinity-purified polyclonal (GR-H300) or monoclonal (GR-G5) antibodies commercially provided by Santa Cruz Biotechnology (Inc, Europe, Heidelberg, Germany) were used. Mouse monoclonal antibodies against -α-tubulin, -BcL-xLs, -pyruvate dehydrogenase (PDH), -citrate synthase, -glyceraldehyde 3-phosphate dehydrogenase (GAPDH), -peroxisome proliferator-activated receptor alpha (PPARα), -mitochondrial transcription factor A (mtTFA), -malate dehydrogenase 2 (MDH2), -lactate dehydrogenase (LDH), -cytochrome c (cyt c), -cytochrome c oxidase assembly protein COX15 homolog (COX15), -mitochondrial cytochrome c oxidase subunit 5B (COX5B), -cytochrome c oxidase subunit 1 (COXI), -cytochrome c oxidase subunit 2 (COX2), -NADH dehydrogenase [ubiquinone] 1 alpha subcomplex subunit 13 (Grim 19 or NDUFA13), -NAD(P)H: quinone oxidoreductase 1 (NQO1), and -Beclin 1 (BECN1), and rabbit polyclonal antibodies against -α-tubulin, -phosphoenolpyruvate carboxykinase (PEPCK), and -p65 subunit of NF-κB were obtained from Santa Cruz Biotechnology (Inc, Europe, Heidelberg, Germany). In addition, mouse monoclonal antibodies against -β-actin (Sigma Aldrich, St. Louis, MO, USA), -succinate dehydrogenase (SDH) (Invitrogen, Thermo Scientific, GmbH, Frankfurt, Germany), -caspase 9 (Cell Signaling Technology, Leiden, The Netherlands), -cytochrome c oxidase subunit 4 (COXIV) (Abcam, Cambridge, UK), and -mitochondrial cytochrome c oxidase subunit 2 (COXII) (Invitrogen, Thermo Scientific, GmbH, Frankfurt, Germany) were used. Rabbit polyclonal antibodies against -caspase 3, -Bcl-2, -Bax, and -BCL2/Adenovirus E1B 19 kDa protein-interacting protein 3-like (BNIP3L) purchased from Cell Signalling Technology (Leiden, The Netherlands), and anti-P62/Sequestome 1 (SQSTM1) and anti-microtubule-associated proteins 1A/1B light chain 3B (LC3B) antibodies obtained from MBC BioLabs (San Francisco, CA, USA) and Abcam (Cambridge, UK), respectively, were also used. Finally, a goat polyclonal antibody against mitochondrial NADH dehydrogenase [ubiquinone] iron-sulfur protein II (NDUFS2) was purchased from Thermo Fisher scientific (GmbH, Frankfurt, Germany). Details on the source of antibodies and dilutions used are presented in Table 1.

### 4.3. Cell Culture-Mitochondrial Isolation

Human hepatocarcinoma HepG2 cells stably transfected to overexpress a mitochondrial-targeted green fluorescence (mtGFP) protein (HepG2mtGFP cells) or a mitochondrial-targeted GFPGR (mtGFPGR) protein (HepG2mtGFPGR cells) were generated in our laboratory [5] and were maintained in DMEM, supplemented with 10% FBS, 2 mM glutamine, and penicillin/streptomycin. Cells were grown at 37 °C in a humidified atmosphere with 5% CO_2_, in 75 cm^2^ flasks. When cells reached 80–90% confluency, cells were washed twice with ice-cold PBS (9.1 mM dibasic sodium phosphate, 1.7 mM monobasic sodium phosphate, 150 mM NaCl, pH 7.4), trypsinized in 0.25% trypsin, followed by cell counting in a hemocytometer to obtain the appropriate number of cells, as indicated below, for mice injection and tumorigenesis studies. Mitochondrial isolation from cells of the produced tumors was performed as previously described [5]. Briefly, for mitochondrial isolation, tumors tissues were weighed, cut into pieces, and resuspended in 4 volumes of homogenization buffer (20 mM Hepes-KOH, pH 7.5, 10 mM KCl, 1.5 mM MgCl_2_, 1 mM EDTA, 1 mM EGTA, 2 mM dithiothreitol (DTT), and 0.1 mM phenylmethylsulfonyl fluoride (PMSF), containing 250 mM sucrose) with addition of protease inhibitors (cocktail from Roche supplemented with 10 μg/mL of chymostatin, 2.5 nM pepstatin, 10 μg/mL of N-p-Tosyl-L-phenylalanine chloromethyl ketone (TPCK), 10 μg/mL of Na-p-Tosyl-L-lysine chloromethyl ketone hydrochloride (TLCK), 20 μg/mL of calpain inhibitor I, 20 μg/mL of calpain inhibitor II), and homogenized at 4 °C, with 20 strokes of a glass Potter–Elvejhem homogenizer, with a Teflon pestle. The homogenate was centrifuged for 5 min at 1000× *g* and the supernatant was collected and further centrifuged at 10,000× *g*, for 20 min. The crude mitochondrial pellet was washed three times in buffer B (20 mM Tris pH 7.5, 0.07 M sucrose, 0.21 M mannitol, 2.5 mM EDTA, 2.5 mM EGTA). Crude mitochondrial pellets were maintained at −80 °C.

### 4.4. Animals

Male NOD/SCID (*n* = 16) and NSG mice (*n* = 20) of 6–8 weeks of age were housed in a specific pathogen-free (SPF) environment under controlled conditions regarding light (12 h cycle), humidity (~50–60%), and temperature (20–22 °C), and allowed food and water ad libitum. The handling and experimentation of the animals were conducted in accordance with the Greek laws (PD 56/2013 and Circular 2215/117550/2013) and the guidelines of the European Union (2013/63/EU) under a licensed protocol approved by the IACUC and Greek authorities (License no. 5542/228006).

### 4.5. Xenografts

Viable (viability > 90% as confirmed with trypan blue dye under an inverted microscope), 1 × 10^6^ hepatocarcinoma HepG2mtGFP or HepG2mtGFPGR cells were injected into the left and right flanks of immunosuppressed NOD/SCID mice (8 mice per group). NOD/SCID mice are homozygous for the severe combined immune deficiency spontaneous mutation (Prkdcscid) and are characterized by an absence of functional T cells and B cells and some Natural Killer (NK) cell functions (normal antigen-presenting cell, myeloid, and NK functions as though background-strain-dependent) [45]. In addition, 20 NSG (NOD scid gamma) mice were inoculated into the posterior axillary area, with 1 × 10^5^ and 1 × 10^4^ HepG2mtGFP or HepG2mtGFPGR cells (5 mice per case). NSG mice due to the scid mutation are deficient for B and T cells while the IL2rg null mutation prevents cytokine signaling through multiple receptors, leading to a deficiency in functional NK cells (triple-deficient mice) [46]. In all cases, the cells were resuspended in plain RPMI medium. Tumor sizes were measured weekly and tumor volume was calculated following the formula: width^2^ × length / 2. At 8 to 9 weeks, mice were euthanized, and tumors were excised, weighted, and cut into pieces. Tumor pieces of approximately (0.2–1.6 mg) were immediately subjected to homogenization and subcellular fractionation to obtain total, post-mitochondrial, and crude mitochondrial extracts. Isolated tumors and cellular and subcellular extracts were maintained at −80 °C.

### 4.6. Electrophoresis and Western Blot

After Bradford protein determination [47], Western blot analysis was performed as previously described [48]. Cellular and subcellular extracts were electrophoresed in discontinuous SDS-PAGE and Western blotted with specific antibodies against proteins of interest as indicated in detail in the “Results” section. β-actin, α-tubulin, GAPDH, or SDH expression levels were evaluated for the normalization of the results. The list of antibodies used and details on the antibodies’ source and working dilution are presented in Table 1. Enhanced chemiluminescence was used for the detection of the protein bands. Quantification of bands intensity was carried out by applying ImageJ (1.52p) analysis (NIH, Bethesda, MD, USA). Background subtraction was performed to assess band intensity. Relative protein levels were expressed as band intensity normalized against the respective band’s intensity of β-actin, α-tubulin, or GAPDH (for total or post-mitochondrial fractions), or SDH (for mitochondrial fractions). Relative protein levels in HepG2mtGFP tumors were set as 1.

### 4.7. Citrate Synthase Enzymatic Assay

Citrate synthase (CS) activity was measured at 37 °C as previously described [49]. Briefly, 20 μL of mitochondria extracts was added to 930 μL of reaction buffer (0.1 M Tris-HCl—pH 8.1, 0.25% Triton X-100, 0.1 mM freshly made DTNB, 0.31 mM acetylCoA). The absorption at 412 nm was followed for 3 min to measure possible acetylCoA deacetylase activity. The citrate synthase reaction was then started by the addition of 50 μL of freshly prepared 10 mM oxaloacetic acid (final 0.5 mM) to initiate the reaction, and absorbance was read at 412 nm in a spectrometer. CS activity was assessed as Units/mg of protein extract. Citrate synthase activity in mitochondrial extracts from HepG2mtGFP-induced tumors was set at 1. Relative citrate synthase activity was expressed as the ratio of HepG2mtGFPGR/HepG2mtGFP citrate synthase activity.

### 4.8. Pyruvate Dehydrogenase Enzymatic Assay

For pyruvate dehydrogenase measurement, 1 V of mitochondrial pellet was diluted in 9 V of 0.05 M potassium phosphate pH: 7.8, containing 1 mM beta-mercaptoethanol, 1 mM EDTA, 0.1% Triton X-100, and 0.1 mM PMSF supplemented with protease inhibitors cocktail. Pyruvate dehydrogenase activity was measured as previously described [50]. Briefly, an INT-coupled spectrophotometric assay measured the absorbance at 500 nm at 37 °C. The reaction mixture contained 2.5 mM NAD, 0.2 mM thiamin pyrophosphate, 0.1 mM coenzyme A, 0.3 mM dithiothreitol, 5 mM pyruvate, 1 mM magnesium chloride, 0.6 mM INT (iodonitrotetrazolium or 2-(4-iodophenyl)-3-(4-nitrophenyl)-5-phenyl-2H-tetrazolium), 10 μΜ PMS (phenazine methosulfate) mitochondrial extract, and 0.1 mg/mL of BSA in 0.05 M potassium phosphate buffer, pH 7.8. All assays were performed by double-beam spectrometry. A base line was determined with sample and reference cuvettes both containing the entire reaction mixture except for pyruvate. To initiate the reaction, pyruvate was added to the sample cuvette and an equal volume of water was added to the reference. Pyruvate dehydrogenase activity was assessed as Units/mg of protein extract. Pyruvate dehydrogenase synthase activity in mitochondrial extracts from HepG2mtGFP-induced tumors was set at 1. Relative pyruvate dehydrogenase activity was expressed as the ratio of HepG2mtGFPGR/HepG2mtGFP pyruvate dehydrogenase activity.

### 4.9. Statistical Analysis

All results are expressed as mean ± SD (*n* = 3–8). Data were analyzed by independent t-test (Figure 4, Figure 5, Figure 6, Figure 7, Figure 8 and Figure 9) using SPSS software (Chicago, IL, USA). In vivo data were analyzed by two-way analysis of variance (ANOVA) followed by Tukey’s post hoc test (Figure 1 and Figure 2) or multiple unpaired t-tests (Figure 3) using the GraphPad Prism software (LLC, Boston, MA, USA). Differences were considered significant at a *p* value < 0.05.

## Figures and Tables

**Table 1:** List of antibodies used in Western blot analyses.

Antibodies	Source	Catalog Number	Working Dilution
α-tubulin	Santa Cruz	SC-9104	1/1000
β-actin	Sigma	A5316	1/4000
Bax	Cell Signaling	2772	1/1000
BcL-xS/L(D-3)	Santa Cruz	SC-271121	1/250
Bcl-2	Cell Signaling	2876	1/1000
Caspase 3	Cell Signaling	9662	1/1000
Caspasae 9	Cell Signaling	9508	1/1000
GR-H300	Santa Cruz	SC-8992	1/1000
GR (G-5)	Santa Cruz	SC-393232	1/1000
PEPCK	Santa Cruz	H300	1/1000
PDH	Santa Cruz	SC-65242	1/1000
SDH	Invitrogen	A11142	1/1000
COX II	Invitrogen	A-6404	1/1000
Citrate synthase	Santa Cruz	SC-390693	1/1000
Grim 19 (F10)	Santa Cruz	SC-365978	1/250
NQO1 (H9)	Santa Cruz	SC-376023	1/250
COX 5b (C-5)	Santa Cruz	SC-374416	1/250
BECN1 (E-8)	Santa Cruz	SC-48341	1/250
GAPDH (G-9)	Santa Cruz	SC-365062	1/100
COX 15 (G10)	Santa Cruz	SC-390987	1/250
PPARα (H2)	Santa Cruz	SC-398394	1/500
MDH2 (1G12)	Santa Cruz	SC-293474	1/250
mtTFA (C-9)	Santa Cruz	SC-376672	1/250
Anti-LC3B	Abcam	Ab48394	1/1000
BNIP3L/NIX (D4R4B)	Cell Signaling	12396	1/1000
P65	Santa Cruz	SC-109	1/1000
LDH	Santa Cruz	Sc-133123	1/250
P62/SQSTM1	MBC	PM045	1/1000
NDUFS2	Thermo scientific	PA5-19342	1/500
COXIV	Abcam	Ab33985	1/1000

## Data Availability

All data, tables, and figures are original. Details on data analysis are available from the corresponding author upon reasonable request.

## Supplementary Materials

The following supporting information can be downloaded at: https://www.mdpi.com/article/10.3390/ijms24043740/s1, Figure S1: HepG2 tumors generated in NOD/SCID mice; Figure S2: HepG2 tumors generated in NSG mice; Figure S3: Assessment of endogenous GR level in HepG2 tumors developed in NOD/SCID and NSG mice. Table S1: List of chemicals, source, and codes.
