# Supplementary material for: Increased Expression of the Mitochondrial Glucocorticoid Receptor Enhances Tumor Aggressiveness in a Mouse Xenograft Model
Source: Int J Mol Sci. 2023 Feb 13;24(4):3740. doi: 10.3390/ijms24043740 (PMC9966287; doi:10.3390/ijms24043740)
Supplement: Supplementary file 1 [file ijms-24-03740-s001.zip › Figure S1.pdf]

Supplementary Figure S1

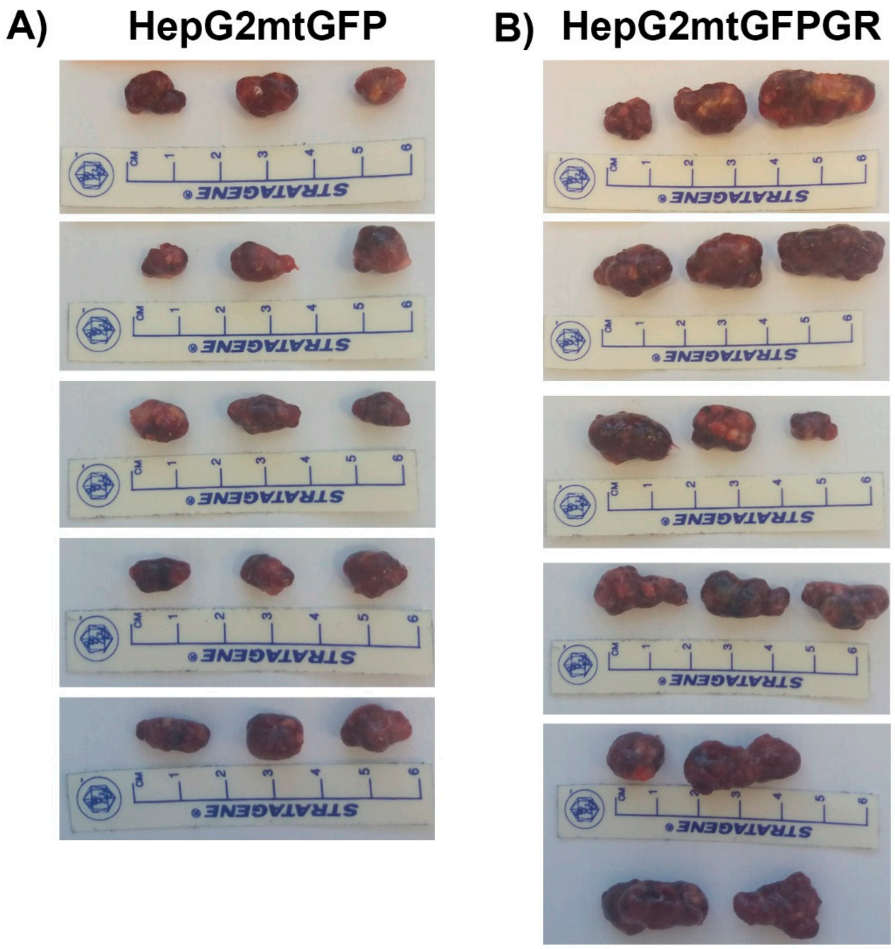

Figure S1. HepG2 tumors generated in NOD/SCID mice. Each mouse was inoculated bilaterally in the axillary regions of the rear flanks with A) $1X10^6$ HepG2mtGFP cells or B) $1X10^6$ HepG2mtGFPGR cells. n=8/group (two tumors/mouse). In the HepG2mtGFP group out of the 16 injections 15 tumors were developed.
