# Supplementary material for: Increased Expression of the Mitochondrial Glucocorticoid Receptor Enhances Tumor Aggressiveness in a Mouse Xenograft Model
Source: Int J Mol Sci. 2023 Feb 13;24(4):3740. doi: 10.3390/ijms24043740 (PMC9966287; doi:10.3390/ijms24043740)
Supplement: Supplementary file 1 [file ijms-24-03740-s001.zip › Figure S2.pdf]

Supplementary Figure S2

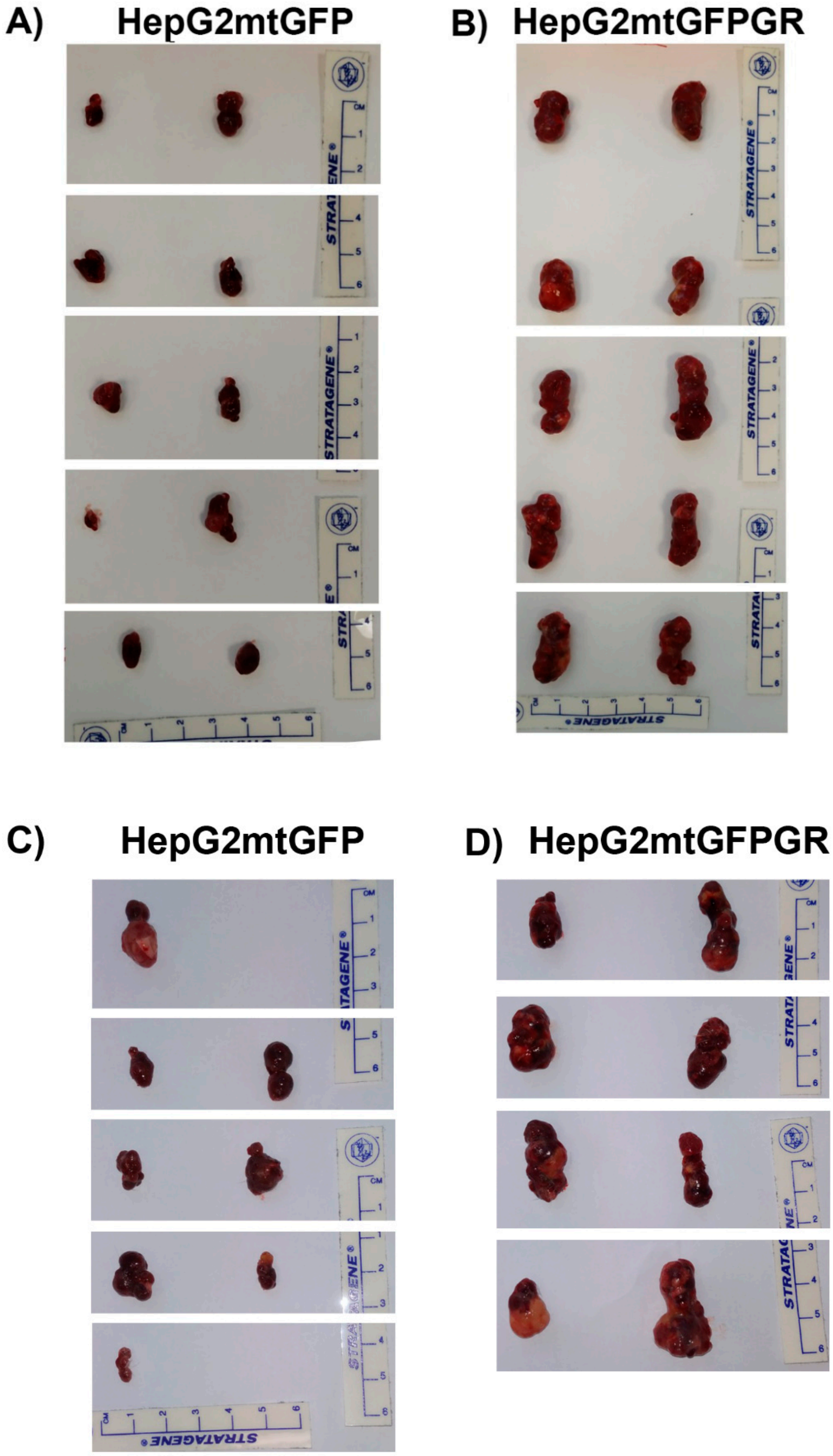

Figure S2. HepG2 tumors generated in NSG mice. Each mouse was inoculated bilaterally in the axillary regions of the rear flanks with A) 1X10^5 HepG2mtGFP cells, B) 1X10^{5}HepG2mtGFPGR cells, C) 1X10^4 HepG2mtGFP cells, D) 1X10^{4}HepG2mtGFPGR cells, n=5 (2 tumors/mouse)/group. In the case of C out of the 10 injections were developed 8 tumors

while in D one mouse found dead on the day of tumors' excision and thus was not included in the postmortem analysis.
