# Supplementary material for: Increased Expression of the Mitochondrial Glucocorticoid Receptor Enhances Tumor Aggressiveness in a Mouse Xenograft Model
Source: Int J Mol Sci. 2023 Feb 13;24(4):3740. doi: 10.3390/ijms24043740 (PMC9966287; doi:10.3390/ijms24043740)
Supplement: Supplementary file 1 [file ijms-24-03740-s001.zip › Figure S3.pdf]

Supplementary Figure S3

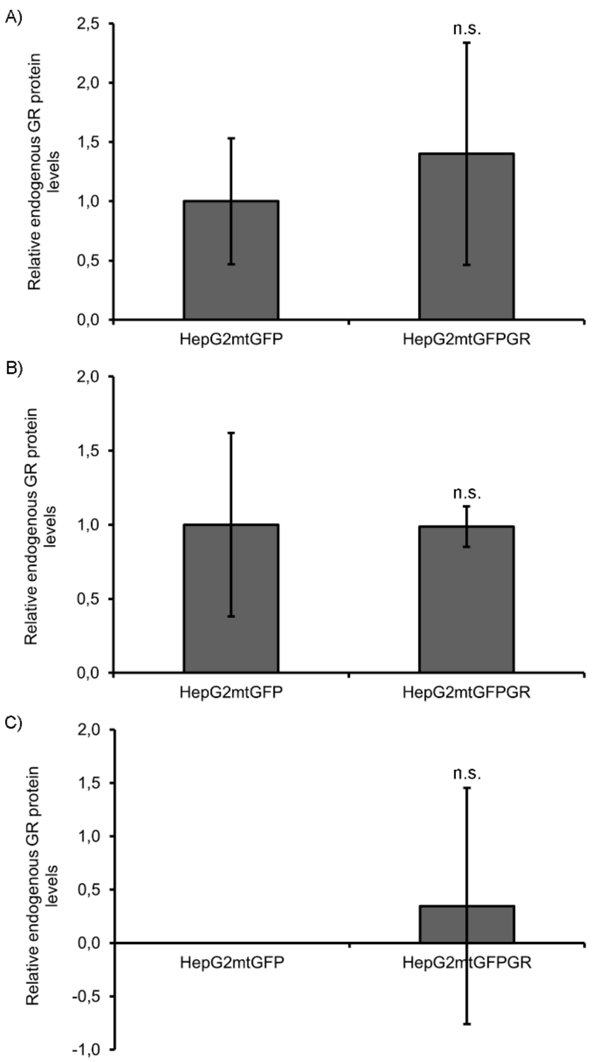

Figure S3. Assessment of endogenous GR level in HepG2 tumors developed in NOD/SCID and NSG mice.

A) Quantification of the endogenous GR level in tumors developed in NOD/SCID mice inoculated with $1x10^6$ of either mtGFPHepG2 cells or mtGFPGRHepG2 cells. Quantification of bands' intensity was carried out by applying ImageJ (1.52p) analysis (NIH, Bethesda, MD, USA) after correcting for the background signal. Relative protein levels were expressed as bands' intensity normalized to the respective intensity of β-actin. Relative protein levels in HepG2mtGFP tumors were set as 1. Data are expressed as mean ± S.D. (n=5). B) Quantification of the endogenous mitochondrial GR level in tumors developed in NSG mice inoculated with $1x10^5$ of either mtGFPHepG2 cells or mtGFPGRHepG2 cells. Quantification of bands intensity was carried out as described above and the relative protein levels were expressed as bands' intensity normalized to the respective intensity of SDH. Relative protein levels in HepG2mtGFP tumors were set as 1. Data are expressed as mean ± S.D. (n=3). C) Quantification of endogenous mitochondrial GR level of tumors developed in NSG mice inoculated with $1x10^4$ of either mtGFPHepG2 cells or mtGFPGRHepG2 cells. Quantification was performed as above. Relative protein levels were expressed as bands' intensity normalized to the respective

intensity of SDH. Data are expressed as mean ± S.D. (n=3). *n.s.,* statistically non-significant compared to the HepG2mtGFP cells (controls).
