# Supplementary material for: Increased Expression of the Mitochondrial Glucocorticoid Receptor Enhances Tumor Aggressiveness in a Mouse Xenograft Model
Source: Int J Mol Sci. 2023 Feb 13;24(4):3740. doi: 10.3390/ijms24043740 (PMC9966287; doi:10.3390/ijms24043740)
Supplement: Supplementary file 1 [file ijms-24-03740-s001.zip › Table S1.pdf]

Table S1

Chemicals	Company (Code)
NAD	Sigma-Aldrich (N0632)
Thiamine pyrophosphate (TPP)	Sigma-Aldrich (C8754)
Iodonitrotetrazolium violet-formazan Crystalline (INT)	Sigma-Aldrich (I7375)
Phenazine methosulfate (PMS)	Sigma-Aldrich (P9625)
MgCL2	Alfa Aesar GmbH (12315)
DTT	BioChemica AppliChem (A1101,0005)
Co-A	AppliChem (A0812,0025)
BSA	Sigma (A9418-10G)
Potassium phosphate	SERVA (26887.02)
5,5-dithio-bis-(2-nitrobenzoic acid) (DTNB)	Sigma-Aldrich (D218200)
Oxaloacetate	Sigma -Aldrich O4126
Acetyl-CoA	Sigma-Aldrich (A2056)
Triton-X 100	Sigma (102233830)
Sodium Pyruvate	Sigma-Aldrich (P2256)

Table S1. List of chemicals, source, and codes
